# The diverse roles of Hox proteins in the regulation of the cell cycle and their therapeutic value in cancer treatment

**DOI:** 10.1007/s10555-026-10313-6

**Published:** 2026-01-15

**Authors:** Lupeuea Vakafua, Lachlan Clifton-Bligh, Naisana Seyedasli

**Affiliations:** 1 School of Medical Sciences, Faculty of Medicine and Health, Westmead Hospital, University of Sydney, Westmead, NSW 2145 Australia; 2 Centre for Cancer Research, The Westmead Institute for Medical Research, Sydney, NSW 2145 Australia

**Keywords:** Hox genes, Cell cycle, Cancer, Differentiation, Disease prognosis

## Abstract

Tissue morphogenesis and homeostasis occur at the interface of a fine coordination between cell cycle and differentiation events. The fundamental role of Hox genes during embryonic development is well established. However, less is known about their role in adult tissues and in contexts other than cellular differentiation. In this review, we focus on the role of Hox proteins in cell cycle-related events in adult tissues and highlight two main cell cycle-permissive and repressive roles for these genes. Given the significance of Hox proteins as a therapeutic target in cancer treatment, we will further discuss the existing approaches in this domain highlighting the challenges that impact the success prospects. For the successful development and application of Hox-oriented anticancer therapies, a comprehensive image of Hox roles in carcinogenic events including their mechanism of function as well as the networks/cascades within which they exert their effects is an inevitable requirement. Moreover, the functional variations of Hox genes in response to changes in the tumour microenvironment along with possible alternations of the mutational landscape in tumours during disease progression should also be taken into account as factors that might impact the therapeutic outcomes of Hox-targeting approaches.

## Overview of hox genes

**Fig. 1:**
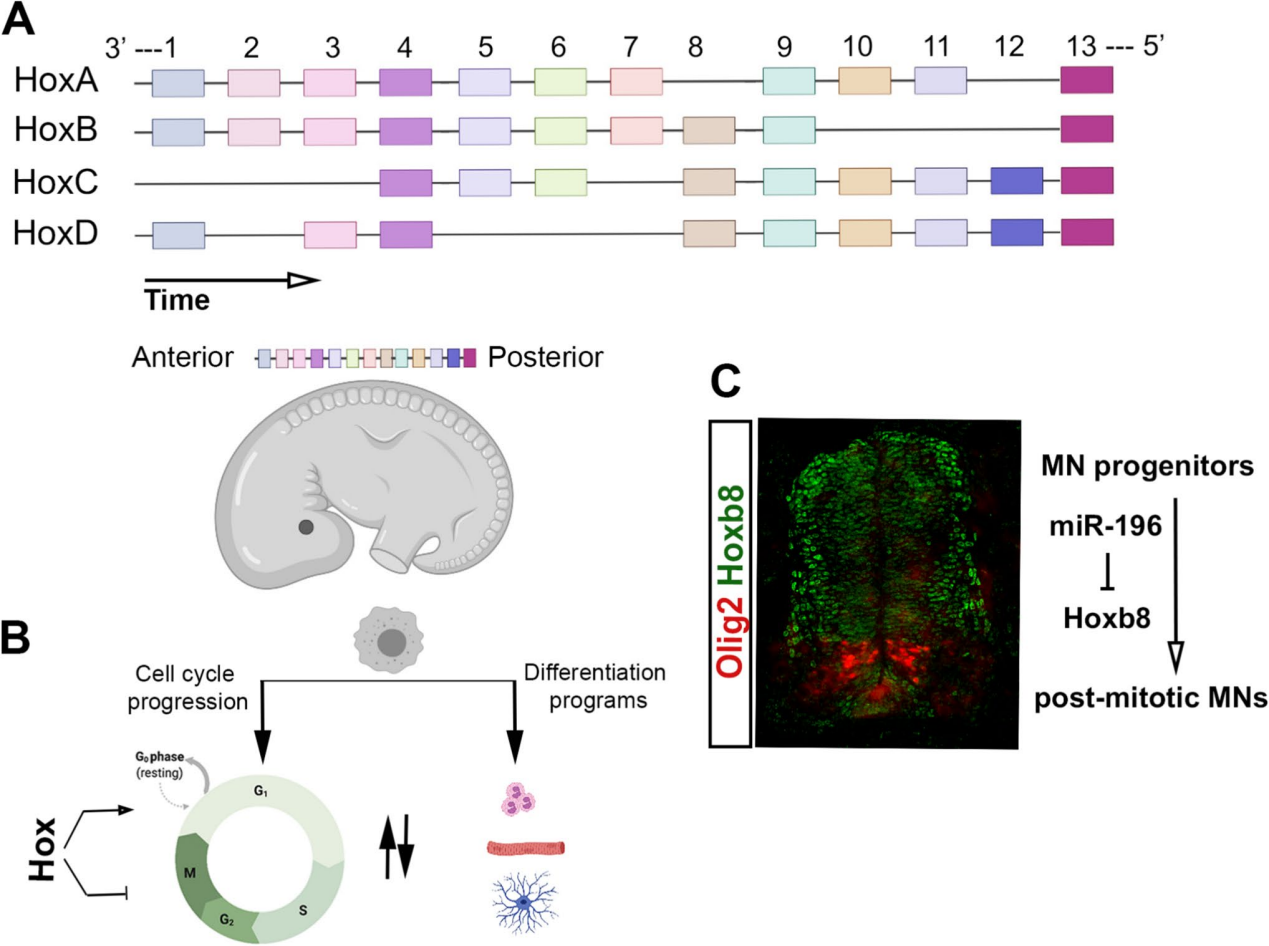
Overview of Hox genes and cell cycle-related events. **A** Structure of human Hox clusters, depicting the concepts of spatial and temporal collinearity. **B** The decision tree between cell cycle progression and differentiation events. **C** Immunohistochemistry on lumbar neural tube of HH22 chicken embryos with antibodies against Olig2 and HoxB8 MN, motor neurons

Embryonic development and patterning occur through complex cellular events with defined molecular regulatory cascades active at key temporal and spatial coordinates. During embryogenesis, genes within the Hox clusters are primarily involved in the early determination of the cell fate territories along the antero-posterior (AP) axis. The 39 highly conserved mammalian Hox genes are organised in four paralogous clusters labelled A, B, C and D spread across four chromosomes [1] (Fig. 1A). It is well established that in vertebrates, the expression of Hox genes follows spatial and temporal collinear patterns where in each cluster, genes located at the 3′ end of the chromosomes, are expressed earlier and more anterior whereas more 5′ genes assume more posterior expression domains that occur later in the developmental timeline [2–4] (Fig. 1A). Hox genes are well described as potent determinants of early embryonic patterning with mutations in humans and/or animal models resulting in severe developmental defects including synpolydactyly, hand–foot–genital syndrome and Charcot–Marie–tooth disease [2, 3, 5, 6]. As embryonic development progresses, the expression patterns of Hox genes reconfigure in alignment with more specific roles for these genes during tissue morphogenesis and specialised differentiation [7, 8].

Although the roles of Hox genes in embryonic patterning are extensively studied, less is known about their contributions to adult organogenesis and disease. Regardless of the developmental window within which Hox genes are active, majority of functions assigned to these genes are related to differentiation pathways [2, 9]. It is well acknowledged that for differentiation cues to initiate, cells should reach a certain degree of readiness at which stage the otherwise cycling cells retract from the cell cycle to initiate the differentiation process. To coordinate these two events, tight molecular machineries are involved in the so-called coupling of cell proliferation and differentiation [10, 11]. As Hox genes are primarily described to function within the differentiation programs, in essence, it is anticipated for these genes to be anti-proliferative. Profiling of Hox gene expressions in human cancers, however, alludes to a much more complex image with upregulation of many Hox genes observed in various types of cancer [12, 13]. In this review, our focus is on the modes of cell-cycle related Hox gene functions, and we will use examples from embryonic development and/or adult homeostasis and disease to define and discuss different modes of action and roles of Hox genes in cell cycle progression, both as inhibitory and permissive factors. Building on this context, we will then critically discuss examples of Hox-targeting anticancer approaches.

### Coupling of cell cycle and differentiation programs

The generation of a multicellular organism requires a fine spatial and temporal coordination between cell proliferation and differentiation. As a general rule, the two processes show an inverse relationship where terminal differentiation is conditional to complete irreversible or partially reversible arrest of the cell cycle [11] (Fig. 1B). The progression through the cell cycle is largely mediated by the formation of phase-specific cyclin–cyclin-dependent kinase (CDK) complexes that in turn initiate specialised downstream phosphorylation events. Upregulation of CDK inhibitors (CKI), the proteins that specifically target and inhibit the formation of cyclin-CDK complexes, is repeatedly reported in several differentiating tissues [14, 15]. The cell cycle entry is as well dependent on the integrity of the specialised cyclins which can in turn be targets for the destruction complexes including a variety of ubiquitin ligases [11]. In fact, differential regulation of Skp2, an E3 ubiquitin ligase, as a regulator upstream of the CKI, P27^kip1^[16], and also other cell cycle factors [17] has been reported in several differentiation pathways.

Direct antagonism between transcription factors involved in differentiation and the CDKs is as well supported by many studies. During the differentiation of C2C12 murine myoblasts to mature skeletal muscles for example, a negative cross regulation between a variety of myogenic transcription factors and the G1 phase CDK4-CyclinD complex has been reported [11]. A similar pattern of cross regulation has been observed during neuronal differentiation, where neurogenic factors like neurogenin 2 (Ngn2) were inhibited by Cyclin-CDK complexes [11, 18]. Studies as well support a pro-differentiation role for the negative regulators of the cell cycle. Direct binding of CKI, P57^kip2^ to the muscle differentiation factor MyoD, for example, is reported to stabilise MyoD and hence promote differentiation [19]. In another example, the tumour suppressor retinoblastoma protein (pRb), was shown to negatively regulate the promyelocytic differentiation program by direct sequestration of the PU.1, the transcription factor involved in the induction of the myeloid lineage [20]. A fine level of coordination between cell cycle progression and differentiation is also mediated by direct transcriptional involvement of lineage specific transcription factors in the expression of cell cycle modulators. In the muscle lineage, for example, MyoD could trigger the expression of CKIs including P57^kip2^ and P21^Cip1^ [21]. The promoter for the gene encoding the P21^Cip1^ CKI is as well targeted by transcription factors of the erythrocyte lineage resulting in the expression of this CKI alongside the onset of the differentiation program [22]. Combined, these mechanisms ensure a fine and well-tuned regulation between programs that govern a cell’s progress through the cell cycle, or the lineage-determined differentiation paths.

### Hox genes could repress the progression through the cell cycle or the cell cycle-associated events

Given the inverse correlation between pathways that define differentiation or cell cycle progression, one would anticipate for Hox genes to have an anti-proliferative role and hence be repressed upon cell cycle activation. In fact, ample evidence is in support of such a role for Hox genes. Several studies have alluded to an anti-proliferative role for multiple Hox genes during neurogenesis in the developing nervous system of the fruit fly, drosophila (*Drosophila melanogaster)*. In fact, morphogenesis and regional differentiation of the drosophila ventral nerve cord occurs in accordance with a finely tuned gradient of at least three Hox genes of the Bithorax (BX-C) complex: Ultrabithorax (Ubx), Abdominal-A (Abd-A) and Abdominal-B (Abd-B), whose distinct expression gradients along the anteroposterior neural axis defines the frequency and locations of cell cycle exit and differentiation in neural progenitors [23]. Single or joint mutants of BX-C complex showed a significant reduction in the expression levels of several cell cycle regulators including Cyclin E, E2F1, String (Cdc25) and Decapo (P21^Cip1^/P27^Kip1^/P57^Kip2^) further confirming a functional interplay between these Hox genes and the cell cycle machinery. Interestingly, the role of Hox-Cyclin E axis has been highlighted in other studies as a neural cell fate determinant. In the developing drosophila central nervous system (CNS), Cyclin E seems to be both necessary and sufficient to induce the neural cell lineage, and once induced by BX-C, it is able to maintain the asymmetric division of neural progenitors leading to balanced differentiation of neuroblasts to neural and glial cells [24, 25]. Although later studies point to a cell cycle-independent function of Cyclin E acting upstream of the glial differentiation factor Prospero to determine the neural vs glial cell fates [26], other cell cycle-related mechanisms for the neurogenic role of Cyclin E and the related networks are yet to be determined.

Conforming to a pro-differentiation role for Hox genes, overexpression of HoxC9 in neuroblastoma cells and xenograft models, resulted in reduced growth potential in these cells and/or tumours [27, 28]. Moreover, further observations alluded to a role for this Hox gene downstream of the retinoic acid (RA)-induced differentiation suggesting a pro-differentiation role for this gene. As such, elevated levels of HoxC9 correlated with improved prognosis and better survival in neuroblastoma patients [29, 30]. HoxC9 was as well shown to play a role in the activation of the intrinsic apoptotic pathway, highlighting an additional role for this protein downstream of cell cycle arrest [30]. In human HeLa cells, a specific mitotic degradation pattern was observed for HoxC10 which provided additional evidence for the requirement of Hox gene downregulation upon progress through the cell cycle [31]. In HeLa cells, a concomitant ubiquitin- mediated degradation of HoxC10 and the late-S/G2 phase cyclin, “Cyclin A”, by anaphase promoting complex (APC) was observed. Subsequently, transfection of the cells with a stable non-degradable HoxC10, resulted in a mitotic cell cycle arrest with a delayed transition from metaphase to anaphase confirming a licensing role for the degradation of this Hox protein during mitosis [31]. Despite the negative role for HoxC10 in cell cycle progression, studies in many cancer types associate elevated levels of HoxC10 with tumour proliferation, metastasis and resistance to treatment, highlighting a multifactorial network of action for this gene in addition to its direct role in cell cycle regulation [32]. An aspect that will be further covered in the next section.

**Fig. 2:**
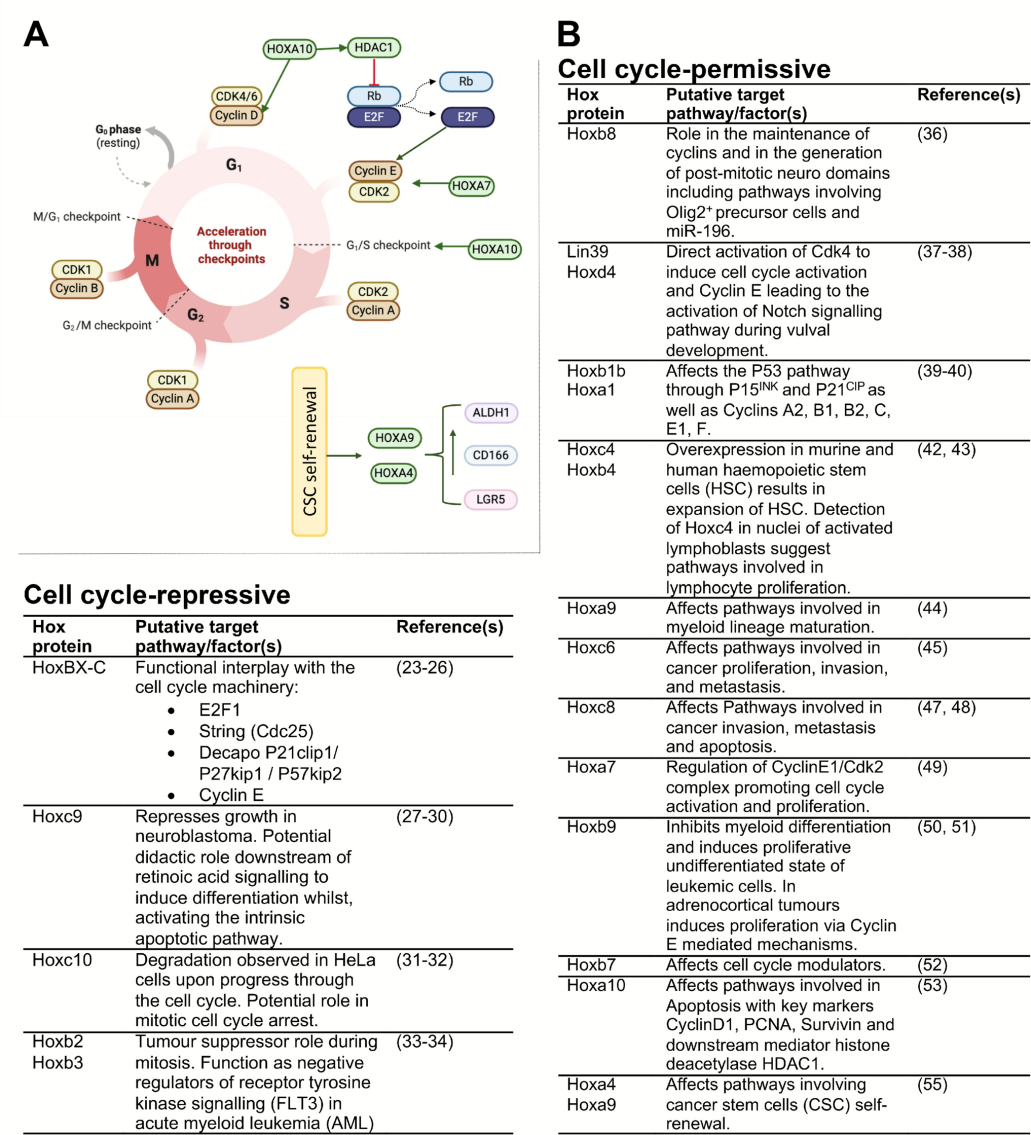
Cell cycle-permissive/repressive effects of Hox genes. **A** Schematic diagram highlighting the effects of Hox genes in the cell cycle and on cancer stem cells (CSC). **B** Detailed summary of Hox genes with permissive and repressive effects on the cells cycle with known pathways affected

Cell cycle-repressive roles of Hox genes has gained additional support from studies in various cancers. In tumour xenografts derived from a range of mammary adenocarcinoma cell lines, HoxB2 was identified as a tumour repressor with changes directly evident in the mitotic index of the target cells [33]. Further, in acute myeloid leukaemia (AML), HoxB2 and HoxB3 were identified as negative regulators of the FLT3 receptor tyrosine kinase, with their mutations associated with carcinogenic events in around 30% of AML cases [34]. Altogether, these studies are in support of a cell cycle repressive role for Hox genes (Fig. 2A and B).

### Hox proteins enhance cell proliferation or specialised aspects of cell division

Despite the role of Hox proteins as anti-proliferative factors, evidence from many cancers demonstrate enhanced expression of Hox genes, implicit of a possible positive role for these proteins in cell cycle events [35]. In fact, even during embryonic development and tissue morphogenesis, Hox genes are found to have cell cycle-permissive roles (Fig. 1B and C). During early generation of lumbar motor neurons, HoxB8 assumes expression domains that are exclusive of Olig2^+^ precursor cells (Fig. 1C). A specific pattern that is further maintained by the complementary function of the HoxB8-targetting microRNA, miR-196. In this context, forced expression of HoxB8 within the precursor motor neuron (MN) domains, or alternatively, inhibition of miR-196, resulted in a significant decrease of post-mitotic MNs. Further, the decline in the abundance of motor neurons was inversely mirrored by an increase in the cycling Olig2^+^ precursor cells highlighting a role for HoxB8 in the maintenance of the cycling state [36]. Studies focusing on *Caenorhabditis elegans (C. elegans)* vulval development, which represents a fine coordination between cell fate determination and cell proliferation, further support the proliferative role of Hox proteins. During vulval development, Lin39, the *C. elegans* equivalent of the mammalian HoxD4, is involved in cell cycle promotion with its downregulation shown to impair the coordinated morphogenic events that lead to vulval fusion [37]. Further studies demonstrated that Lin-39 on one hand induces the cell cycle through direct activation of Cdk4 and Cyclin E, and on the other hand, activates Notch signalling in neighbouring vulval progenitor cells which in turn promotes cycling events [38]. Additional insight to support a cell cycle specific role for Hox proteins is drawn from studies of zebrafish hind brain development. During the morphogenesis of zebrafish hind brain neural tube, HoxB1b, which shares functional similarities with the mammalian HoxA1, is dispensable for the proliferation of neural progenitors and their apical-basal polarity. However, a specialised role was described for this Hox protein in the correct orientation of the mitotic spindles in precursor cells that directly impacts their downstream differentiation heritage and results in significant morphological defects in HoxB1b mutant embryos [39]. In a further study, siRNA-mediated knockdown of HoxA1 (the mammalian equivalent of HoxB1b) in HeLa cells and lung fibroblasts, resulted in a significant downregulation of genes involved in S, G2/M and the mitotic phase of the cell cycle. As well, numerous genes involved in the P53 pathway, including P15^INK^ and P21^CIP^ were upregulated while a significant reduction in the expression levels of Cyclins A2, B1, B2, C, E1 and F was observed [40]. The role of Hox genes in the interface of specialised cell division properties and stem/progenitor cell proliferation, has as well been demonstrated in the developing drosophila testis. In the developing larval testis, loss of Abd-B in germline stem cells (GSC) resulted in perturbed positioning of these cells within the niche, loss of proper centrosome alignment and subsequent impaired cell division within the mutant GSCs [41].

Studies in murine and human haemopoietic stem cells (HSC) highlight a key role for the paralogue Hox genes, HoxC4 and HoxB4, with their overexpression resulting in the expansion of HSCs through similar downstream molecular effectors [42]. Moreover, the detection of HoxC4 protein in the nuclei of activated lymphoblasts further suggests a potential role for this specific Hox protein in lymphocyte proliferation [43]. Similar outcome was reported in HoxA9-deficient HSCs, where despite the presence of normal immune phenotypic stem cells, HoxA9^−/−^ cells showed a significant decline in their growth, multilineage differentiation and repopulating potential [44]. Slowed proliferation was further accompanied by delayed generation of committed progenitors and myeloid maturation both of which effects were rescued upon forced retroviral HoxA9 expression.

Study of Hox genes in human cancer, also alludes to a key role for these proteins as cell cycle mediators. In gastric cancer cells, the expression of HoxC6 associated with cell proliferation, invasion and metastasis [45]. Likewise, in prostate cancer, elevated levels of HoxC6 correlated with tumour proliferation, while knockdown of HoxC6 resulted in marked reduction in the proliferative index of prostate cancer cell lines [46]. Further evidence from this cancer, confirmed that enhanced expression levels of HoxC8 correlated with loss of differentiation suggesting a role for this Hox protein in tumour invasion and metastasis [47]. Likewise, in ovarian epithelial carcinoma, elevated levels of HoxC8 was reported in tumour samples with poor prognosis [48]. Further analysis using siRNA-mediated knockdown of HoxC8 in ovarian carcinoma cell lines confirmed a marked decrease in proliferation and invasion and induced apoptosis [48]. In hepatocellular carcinoma, HoxA7 promoted cell cycle progression and proliferation by regulating the CyclinE1/Cdk2 complex [49]. In a different study, overexpression of HoxB9 was shown to inhibit myeloid differentiation and instead induce the proliferative undifferentiated state of leukaemic cells [50]. The same protein was also found to enhance cell proliferation in adrenocortical tumours presumably through a Cyclin E-mediated mechanism [51]. In breast cancer, HoxB7 associated with high proliferative index with many direct target genes from cell cycle modulators providing another example of a cell cycle-permissive role for Hox proteins [52]. In hepatocellular carcinoma, knockdown of HoxA10 resulted in cell cycle arrest at G0/G1 phase of the cell cycle with a downstream apoptotic outcome, concomitant with a reduction in markers of cell cycle activity, including cyclin D1, PCNA and survivin [53]. Further investigations also highlighted a key role for the histone deacetylase HDAC1, as a downstream mediator of HoxA10 with similar effects on proliferation, cell cycle progression and apoptosis [53]. Although direct effects of Hox proteins on the cells cycle have been demonstrated in many contexts, it is also worthwhile to consider common mediators that simultaneously and often in parallel modulate the cell cycle and Hox gene expression. One such factor is the protein coded by the mixed lineage leukaemia gene (MLL). Originally isolated as a gene with frequent translocations in leukaemia, this human orthologue of the *Drosophila trithorax*, is a histone methyltransferase. These translocated gene loci generate chimeric fusion proteins with regulatory functions upstream of a number of Hox cluster genes.

Apart from overall effects of Hox proteins on cancer cell proliferation, evidence also supports a role for these proteins in specialised cell populations within tumours known as cancer stem cells (CSC) (Fig. 2A). CSCs are stem-like cells identified in many cancer models with key roles in cancer initiation, progression, metastasis and resistance to treatment [54]. As an example, expression of Hox genes in the CSC compartment is reported in colorectal cancer, where overexpression of HoxA4 and HoxA9 induced CSC self-renewal and overpopulation. Further analysis confirmed that reduced expression of the two Hox genes led to a decrease in proliferation and colony formation while a marked decline in the levels of CSC-specific surface markers such as ALDH1, CD166 and LGR5 was observed [55].

Altogether, the studies above support key roles for Hox proteins in cell proliferation and/or cell cycle-specific events (Fig. 2 A and B). Given the dual roles for Hox genes both as mediators of differentiation and cycling events, it might be hypothesised that these proteins are involved in fine coordination of cellular fates and are indeed major players in the coupling machinery between cell cycle and differentiation programs. Further experimental evidence to support this hypothesis however is yet to be provided.

### Targeting the cell cycle effect of Hox proteins as a therapeutic angle in cancer treatment

The common pattern of Hox gene dysregulation in different cancers, plus the experimental evidence supporting a functional cell cycle–centric role for these proteins, marks them as potential targets for cancer treatment. As such, a range of approaches have been trialled to functionally interfere with the Hox-mediated carcinogenic cascades.

Many Hox proteins are functionally dependent on interaction with defined cofactors to facilitate their binding to downstream target genes/promoters or for their trafficking or translocation into the nucleus. An example of such co-factor is the pre-B cell leukemia transcription factor (PBX). PBX is a member of the “Three Amino Acids Loopes Extension” (TALE) class of Homeodomain (HD) proteins. The interaction between Hox and PBX proteins depends on a short motif within the Hox protein known as the hexapeptide (HX), which is located upstream of the HD-containing transcription factors. This HX motif is characterised by an invariant tryptophan (Trp) residue and is present in all paralog groups 1–10 of Hox proteins. The Trp residue interacts with additional residues of the HD bound to PBX when forming a complex with Hox, thereby strengthening the protein–protein contact zones [56].

Formation of the Hox-PBX dimer is essential for nuclear localisation of the Hox proteins and the downstream modulation of the target genes [57]. It was therefore imperative to use the Hox-PBX dimerisation as a target point for functional inhibition of relevant Hox proteins. Along these lines, the HXR9 peptide was trialled as an inhibitor of the dimerisation step. In ovarian cancer cell lines with elevated expression of multiple Hox transcripts across all four clusters, treatment with this peptide induced apoptosis *in vitro* and reduced tumour xenograft growth *in vivo* [58]. Likewise, in breast cancer cells, treatment with HXR9 led to cellular apoptosis. Further analysis also confirmed that the intensity of the reported apoptotic outcome correlated to the extent or levels of HoxB1-b9 gene expressions in the tested cell lines [59]. It is known that in oesophageal squamous cell carcinoma (ESCC), the expression of HoxB7, HoxC6 and HoxC8 correlates with poor prognosis with all three proteins acting as pro-proliferative and anti-apoptotic factors [60]. Although the functional redundancy among the three Hox genes complicates the conventional single-gene targeting approaches, treatment with the HXR9 peptide in ESCC cells effectively interrupted the activation of downstream target pathways with anti-tumour effects evident at the levels of cell proliferation, apoptosis and tumour growth [60]. Further studies in human mesothelioma, where a significant dysregulation of Hox genes is reported, also confirmed an efficient anti-tumour function for the HXR9 peptide [61].

Examples of other DNA-binding interaction partners of Hox proteins allude to additional potential targets for a similar therapeutic strategy. Among these are other members of the TALE class, including the myeloid ecotropic integration site (MEIS) proteins that act within the Hox–PBX complex as functional facilitators. PBX in the Hox–PBX complex forms a stable heterodimer with MEIS1, enabling the complex to translocate to the nucleus and bind to DNA [62]. Expressed in the bone marrow, MEIS1 plays a role in regulating growth and differentiation during embryonic and adult haematopoiesis [63]. This function becomes particularly important in acute leukemia where dysregulation of haematopoeietic progenitor cells promotes uncontrolled proliferation and cancer [64]. In fact, analysis of HoxA9 and MEIS1 gene expression in children with leukemia confirmed the association of both HoxA9 and MEIS1 overexpression in different leukemia subtypes including acute myeloid leukemia (AML) and mixed lineage leukemia (MLL) [65].

In AML, where dysregulation of MEIS1 and HoxA9 is repeatedly reported, forced co-expression of the two proteins significantly enhanced the tumourigenic properties of HoxA9, further highlighting a cooperative effect [66]. Upregulation of MEIS1 in cancers such as breast, colorectal, oesophageal, neuroblastoma, ovarian and prostate has been observed to be associated with increased cancer aetiology and progression [62]. An emerging therapeutic approach includes indirectly targeting the levels of MEIS1 through the epigenetic regulation of Hox9 protein expression [67, 68]. Another epigenetic modulator of HoxA9 and MEIS1 is related to the histone methyltransferase activity of DOT1L [69]. This regulatory relationship has resulted in MLL treatment strategies that selectively inhibit DOT1L hence decreasing the levels of HoxA9 and MEIS1 [70, 71]. An example of such DOT1L inhibitors is pinometostat, EPZ-5676, which has resulted in promising outcomes in paediatric and adult patients with MLL.

Further, the formation of the MEIS-PBX complex, was shown to be essential for the maintenance of protein stability. Experimental interference with the dimerisation between PBX3 and MEIS1 resulted in destabilisation of MEIS1 and a significant decline in HoxA9-induced proliferation and colony formation in primary cells and animal models of leukemia [72].

These studies propose an effective Hox-targeting therapeutic strategy by disrupting the functional Hox complexes and functionally impairing their cascade of action. Of note, specialised motifs distinct from HX are known to mediate the formation of Hox–MEIS–PBX complexes, which could, in turn, provide additional targets with therapeutic values.

Hox genes are regulated by many non-coding transcripts [36]. As such, a valid line of potential therapeutics has formed around the use of Hox targeting non-coding RNA molecules. Hox clusters in fact harbour microRNA coding sequences that could potentially be used to target Hox transcripts [73]. As small 20–22 nucleotides in length of non-coding transcripts, microRNAs (miR) bind to partially complementary sequences commonly in the 3′ untranslated region (UTR) of target mRNAs and result in the inhibition of transcription and/or translation of the prospective mRNAs [74]. In fact, 30 of the 39 Hox genes have conserved nucleotide sequences targeted by miRNAs in vertebrates [75]. These miRNAs seem to mainly regulate Hox genes situated on the 3′ side of their locus along the Hox cluster [73]. The two microRNA families, miR-10 and miR-196, are examples of miR sequences within the Hox clusters [65]. The locations of miRs in Hox genes are relative to the transcriptional orientation of the cluster as seen in miR-10a residing upstream of HoxB4 and miR-10b upstream of HoxD4 [76]. In zebrafish, miR-10 targets HoxB1a and HoxB3a, and miR-10 knockdown leads to HoxB1a and HoxB3a upregulation in the spinal cord [77]. Additionally, miR-10c, encoded within a HoxB3a splice variant, functions in an autoregulatory manner. It has been speculated that Hox gene clustering necessitates post-transcriptional silencing to refine gene expression domains within these densely packed regions [77].

Experimental evidence confirms HoxA1, HoxA3 and HoxD10 as potential targets for the miR-10 microRNA family [76]. Also, for miR-196, HoxB8 and HoxB9 were identified as target Hox transcripts among others [36, 78]. In fact, in clear-cell renal cell carcinoma, miR-10b-mediated downregulation of HoxA3 resulted in the suppression of cell invasion and metastasis highlighting the potential for this and other microRNAs as a regulator of Hox-related carcinogenic events [79]. The fact that the partial complementarity is sufficient for the microRNA target recognition and function heavily extends the breath of potential targets, as well as possible off-targets, for these molecules. Further, the genomic instability and the propensity for *de novo* mutations in cancer cells could potentially alter the microRNA target sequences and introduce variations at the level of off-target events. All facts should be cautiously considered in the use of microRNAs as therapeutic Hox-targeting agents. In addition to their application in suppressing Hox genes as anti-cancer therapeutics, microRNAs within the Hox clusters could also serve as potential cancer biomarkers and predictive tools for various carcinogenic events, as well as in cancer diagnostics. In gastric cancer, the miR-10 family of microRNAs (miR-10a and miR-10b) plays key roles in the regulation of cell cycle at both ends of cell proliferation and apoptosis, as well in cancer cell migration, invasion, metastasis and drug resistance [80]. Similar roles have also been described for miR-10 in other cancers to an extent where it has been highlighted as a predictive biomarker for a variety of cancer properties and/or response to a range of therapeutic approaches. In acute myeloid leukemia (AML), for example, where elevated expression of miR-10a is reported, the levels of this microRNA serve as a predictive biomarker for sensitivity to the emerging mouse double minute 2 (MDM2) inhibition therapy which in turn reactivates the already functional but suppressed P53 [81]. Like miR-10, the other microRNA from the Hox cluster, miR-196 has as well gained attention as a predictive biomarker for a variety of cancer properties. In non-small cell lung cancer (NSCLC), for example, elevated levels of miR-196b correlated with enhanced cancer cell migration and invasion, with further functional gain and loss of function analyses confirming a direct role for this microRNA in either processes [82]. As such, in gastric cancer as well as many others, miR-196 is strongly suggested as a biomarker, predictive of aggressive clinicopathological status which likely functions via modulating a range of target genes and pathways including AK strand transforming (AKT), P27^kip1^ and Hox genes among others [83]. Apart from the two well-described Hox microRNAs mentioned above, other studies have also identified a long non-coding RNA (lncRNA) transcribed from the genetic location between HoxC11 and HoxC12. This lncRNA, known as the Hox transcript antisense RNA (HOTAIR), was shown to influence the transcription of a number of posterior Hox genes, including those of the HoxD cluster, via altering the chromatin accessibility states [84]. Along with dysregulated expression of its target transcripts in human cancers, HOTAIR was as well repeatedly reported to be overexpressed highlighting a role for this lncRNA in carcinogenesis or related cancer-associated events. In colon cancer, for example, enhanced expression of HOTAIR, HoxB13 and HoxC13 was associated with lymph node metastasis [85]. Likewise, a meta-analysis in cervical cancer confirmed a strong association between the elevated levels of HOTAIR and the rate of occurrence, severity and metastasis in patients [86].

Given the diverse cancer-promoting roles of Hox genes in a number of cancer models, therapeutic strategies aiming to directly target and inhibit Hox transcripts or proteins have retained a central focus. However, structural complexity, accessibility and the nuclear location of the Hox proteins have challenged the application of blocking monoclonal antibodies. In fact, novel immunotherapy approaches have focused on the use of nanotechnology in developing blocking immune moieties that facilitate entrance to the cell nucleus or more importantly can transition cross-anatomically thus tackling structures like the blood–brain barrier. The functional redundancy between paralogous genes/proteins has also posed constraints over sequence-guided therapeutic designs like synthetic small interfering RNAs (si-RNA) [87]. Despite this fact, many Hox-targeting si-RNAs have been trialled as potential anti-cancer therapies with some levels of success [87]. The mechanism of RNA interference relies on either the introduction of long double-stranded RNA (dsRNA) into the cytoplasm to be cleaved by the endocuclease Dicer into siRNAs, or by transfecting ready siRNA into the cell. Either ways, the siRNA incorporates into the RNA-induced silencing complex (RISC) where Argo-2 protein in the RISC degrades the sense strand. Target mRNA is then recognised by the remaining antisense strand. Activated RISC-siRNA complex binds and degrades target mRNA resulting in the silencing of the target gene [88]. Models, such as the study conducted by Zhang and associates, suggest effectiveness in siRNA targeting of TUG1 in regulating HoxB7 expression [89]. However, several barriers challenge clinical application of si-RNA-targeted therapies, including cellular uptake, poor half-life, off-target effect, immune stimulation and side effects of packaging-related methods [90–95].

Hox genes often exert their carcinogenic impacts via mediator factors and act within a network to promote or affect cancer-associated properties. As such, therapeutic strategies have achieved successful outcomes through breaking the functional Hox cascades by inhibiting known mediators and/or cytokines involved in the Hox-mediated carcinogenic events. In hepatocellular carcinoma (HCC), overexpression of HoxB5 transactivates the expression of downstream proteins, fibroblast growth factor receptor (FGFR) 4 and C-X-C motif chemokine ligand (CXCL) 1, leading to enhanced HCC metastasis [96]. Interestingly, treatment of HCC cells with either of the small molecule inhibitors BLU-554 or SB265610, which respectively inhibit FGFR4 or CXCL1, significantly impaired the metastatic cascade initiated by the HoxB5 overexpression [96]. As anticipated, expression of either proteins individually or in combination, correlated to poor prognosis in patients, further confirming the three factors to function within the same network [96]. In a different study using the human ovarian adenocarcinoma cells (CAOV-3), overexpression of HoxA4 resulted in anti-migratory effects through the induction of β1 integrin, highlighting this as a potential Hox cascade with therapeutic benefits [97]. In a separate study of HCC, induction of HoxC10 downstream of interleukin-1β (IL-1 β) resulted in reduced tumour encapsulation leading to increased tumour-nodule-metastasis (TNM) stage and poor prognosis [98].

Further analysis confirmed that, in fact, downregulation of HoxC10 significantly reduced IL-1 β-mediated metastasis marking another example of a Hox cascade with potential therapeutic application [98]. As anticipated, in the same study, daily administration of anakinra, the antagonist for the IL-1 β receptor (IL-1R1) resulted in a significant improvement of survival times [98]. More evidence in support of Hox-mediated carcinogenic cascades was derived from studies in colorectal cancer (CRC) where elevated expression of HoxA13 was positively correlated with distant metastasis, higher tumour stage and poor prognosis [99]. Further analysis confirmed the presence of insulin-like growth factor (IGF)−1 receptor as a functional component downstream of HoxA13-mediated metastasis. Inhibition of which by linsitinib significantly impaired the metastasis-associated events that were induced by HoxA13 [99]. In another study in CRC, the mechanism of HoxB5-induced metastasis was investigated where its elevation positively correlated with metastasis to distant organs, progression in tumour stage and poor prognosis [100]. Additional investigations demonstrated that HoxB5 induces invasion and metastasis by transactivating and engaging the related genes C-X-C motif chemokine receptor (CXCR) 4 and the integrin subunit β1 (ITGβ1). Similarly, in this context, inhibition of CXCR4 by AMD 3100 significantly impaired the HoxB5-mediated metastatic cascade [100].

Altogether, the above findings mark the Hox genes/proteins and the related molecular networks as potential therapeutic targets in anticancer treatments.

## Conclusion

As described, Hox proteins have diverse cell cycle-permissive and repressive functions. Of note, the segregation of individual Hox genes between these two distinct categories of events is independent of classical Hox collinear patterns, and either anterior or posterior Hox genes could attain specialised cell cycle-specific roles. As discussed, there is a prominent representation of Hox genes with roles in cellular differentiation processes. However, evidence supports key role for the Hox proteins in cell proliferation and/or specialised cell cycle events where they function within complex cellular and molecular networks.

As highlighted, overrepresentation and commonly misregulated expression patterns of Hox genes in a broad range of cancers, pin them as potential therapeutic targets. One major challenge to single-gene targeting approaches for these genes, however, is the excessive levels of redundancy between Hox genes and, in particular, the paralogous Hox groups. As such, approaches that target functional complexes commonly used among Hox groups have attracted attention. The functional molecular cascades within which Hox genes exert their effects have as well posed a promising avenue for the development of anti-cancer therapeutics. In fact, a thorough understanding of these and other cascades within which the Hox genes exert their functions is essential for the correct determination, design and targeting of Hox-oriented anticancer approaches. The functional dynamics of Hox genes/proteins should be further considered within the changing tumour microenvironment, the unstable mutational landscape of the tumours, and the therapy-induced tumour remodelling. In this context, treatment regimens and methods should align with the real-time image of Hox gene dynamics and profiles along the lifetime of a tumour to achieve the optimal outcomes.

## Data Availability

No datasets were generated or analysed during the current study.
